# Quantitative structure-activity relationship of molecules constituent of different essential oils with antimycobacterial activity against *Mycobacterium tuberculosis* and *Mycobacterium bovis*

**DOI:** 10.1186/s12906-015-0858-2

**Published:** 2015-09-23

**Authors:** Sergio Andrade-Ochoa, Guadalupe Virginia Nevárez-Moorillón, Luvia E. Sánchez-Torres, Manuel Villanueva-García, Blanca E. Sánchez-Ramírez, Luz María Rodríguez-Valdez, Blanca E. Rivera-Chavira

**Affiliations:** Facultad de Ciencias Químicas, Universidad Autónoma de Chihuahua, Circuito Universitario S/N. Campus Universitario II, Chihuahua, 31125 Chih. México; Escuela Nacional de Ciencias Biológicas, Instituto Politécnico Nacional. Prolongación de Carpio y Plan de Ayala S/N, Col. Santo Tomas 11340, México, DF México; Asociación de Jubilados de la Universidad de Guanajuato, Guanajuato, Gto. México

**Keywords:** Essential oils, Terpenes, Phenylpropanoids, QSAR, Antimycobacterial activity

## Abstract

**Background:**

Essential oils and their constituents are commonly known for their antibacterial, antifungal and antiparasitic activity, and there are also reports on the antimycobacterial properties, but more experimental data are needed for the description of the mechanism of action or structural (and molecular) properties related to the antimicrobial activity.

**Methods:**

Twenty-five constituents of essential oils were evaluated against *Mycobacterium tuberculosis* H37Rv and *Mycobacterium bovis* AN5 by the Alamar Blue technique. Twenty compounds were modeled using *in silico* techniques descriptor generation and subsequent QSAR model building using genetic algorithms. The *p*-cymene, menthol, carvacrol and thymol were studied at the quantum mechanical level through the mapping of HOMO and LUMO orbitals. The cytotoxic activity against macrophages (J774A) was also evaluated for these four compounds using the Alamar Blue technique.

**Results:**

All compounds tested showed to be active antimicrobials against *M. tuberculosis*. Carvacrol and thymol were the most active terpenes, with MIC values of 2.02 and 0.78 μg/mL respectively. Cinnamaldehyde and cinnamic acid were the most active phenylpropanes with MIC values of 3.12 and 8.16 μg/mL respectively. The QSAR models included the octanol-water partition (LogP) ratio as the molecular property that contributes the most to the antimycobacterial activity and the phenolic group (nArOH) as the major structural element.

**Conclusions:**

The description of the molecular properties and the structural characteristics responsible for antimycobacterial activity of the compounds tested, were used for the development of mathematical models that describe structure-activity relationship. The identification of molecular and structural descriptors provide insight into the mechanisms of action of the active molecules, and all this information can be used for the design of new structures that could be synthetized as potential new antimycobacterial agents.

## Background

Tuberculosis is an infectious disease caused by *Mycobacterium tuberculosis* and other mycobacteria including *Mycobacterium bovis*. It is estimated that one-third of the world population is infected with *M. tuberculosis* and 5–10 % of them will develop clinical symptoms [1]. The increase in the incidence of clinical tuberculosis is associated with increasing reports of new cases of multi drug resistant (MDR-TB) and extensively multidrug resistant (XDR-TB) strains [2]. The development of new drugs is critical for the future control of tuberculosis (TB) and a number of promising compounds are currently in the pipeline at various stages of drug discovery and clinical development [3]. Under this scenario, the design and synthesis of new anti-TB agents is essential for the development of novel pharmaceutical therapies.

Mexico possesses a vast geographic diversity, and has one of the richest flora on the planet [4]. In Mexico there are more than 4000 species of medicinal plants [5] and many of them produce essential oils, which are water-insoluble highly volatile extracts obtained by hydrodistillation. Their composition is complex and variable, but they are usually terpenes and/or phenylpropanes [6, 7]. Due to their biological properties, including antimicrobial action, there is an increasing interest on terpenes and others molecules present in essential oils [8]; the biological activity varies depending on the structural configuration and functional groups of their constituent [9]. Regarding antimycobacterial activity of terpenes and phenylpropanes, several reports have shown that chain free monoterpenes have antimycobacterial activities of pharmaceutical importance [10–12]. These results have motivated the research on the molecular and reactivity properties of them, as the base for the design of new pharmacologically active compounds, that could use the molecules present in essential oils as building blocks.

Computer assisted prediction of the biological activity in relation to the chemical structure of a compound is a commonly used technique in drug discovery [13, 14]. Quantitative Structure-Activity Relationship (QSAR) studies have been widely used to understand the relationship between the chemical structure and biological activity of the molecules [15]. Our group has previously demonstrated that essential oils have antibacterial activity [16] and antimycobacterial activity against multi drug resistant strains [17]. On the basis of those results, the main objective of this study was to evaluate the antimycobacterial activity of 25 constituent molecules of essential oils in order to obtain a QSAR model that provide information on the elucidation of molecular properties of biological importance. In this context the models will help on the rational design and subsequent synthesis of new antimycobacterial compounds.

This study included the evaluation of the antimycobacterial activity of 25 compounds, terpenes and phenylporpanes that are constituent of different essential oils, against *M. tuberculosis* (H37Rv) and *M. bovis* (AN5) using the Alamar Blue technique. Isoniazid and rifampicin were used as controls. QSAR models were obtained using genetic algorithms techniques, with the inclusion of the four descriptors that had the higher contributions to the antimycobacterial activity.

## Methods

### Bacterial cultures, growth conditions and bactericidal assays

**Fig. 1:**
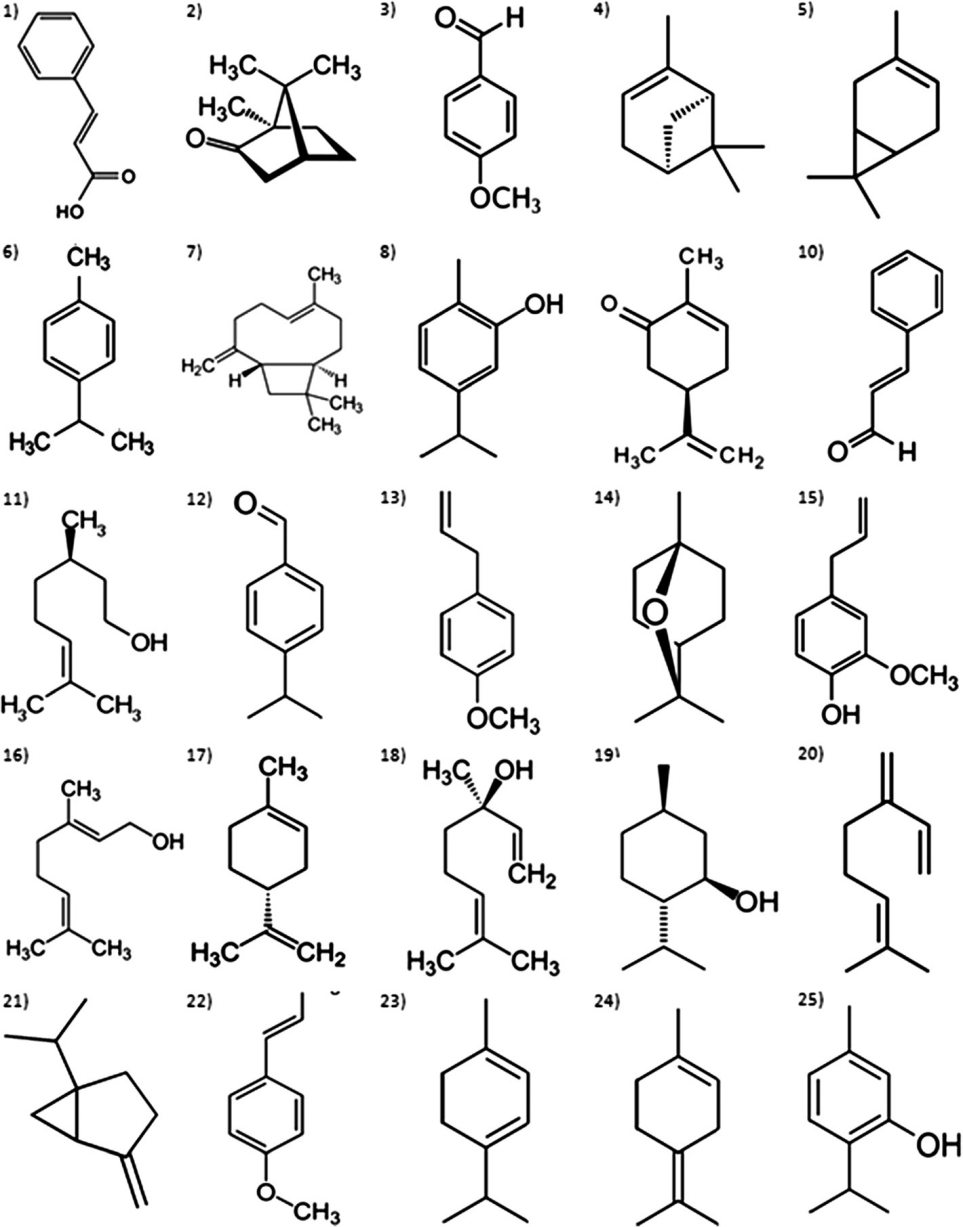
Chemical structures of terpenes and phenylpropanes evaluated against *Mycobacterium tuberculosis* and *Mycobacterium bovis* and studied by molecular modeling technique. 1) Cinnamic Acid; 2) Camphor, 3) *p*-Anisaldehyde; 4) β-pinene; 5) 3-Carene; 6) *p-*Cymene; 7) β-Cariophylene; 8) Carvacrol; 9) (+) Carvone; 10) Cinnamaldehyde; 11) β-Citronellol; 12) Cuminaldehyde: 13) Estragole; 14) Eucalyptol; 15) Eugenol; 16) Geraniol; 17) (+) Limonene; 18) Linalool; 19) Menthol; 20) Myrcene; 21) Sabinene; 22) *t-*Anethole; 23) α-Terpinene; 24) Terpinolene; 25) Thymol

The bacterial strains used in this study were H37Rv strain of *M. tuberculosis* and AN5 strain of *M. bovis*, both obtained from the National Institute of Medical Sciences and Nutrition “Salvador Zubirán” and INIFAP respectively. For the *in vitro* studies all strains were grown in Difco 7H9 Middlebrook liquid media (BD Biosciences, 271310) supplemented with 10 % Middlebrook OADC Enrichment (VWR, 9000–614), 0.05 % Tween (G-Biosciences, 786–519), and 0.2 % Glycerol at 37 °C. *M. tuberculosis* was grown on Difco Middlebrook 7H11 agar (BD Biosciences, 283810) supplemented with 1 % Asparagine. Isoniazid (Sigma, I3377) and rifampicin (Sigma, R3501) were used as controls. The Minimum Inhibitory Concentration (MIC) of 25 major components of different essential oils was evaluated on the H37Rv strain of *M. tuberculosis* and AN5 *M. bovis* strain by the Alamar Blue technique [18, 19]. A growth control containing no antimicrobial compounds and a sterile control were also prepared on each plate. Sterile water was added to all perimeter wells to avoid evaporation during the incubation. The plate was covered, sealed in plastic bags and incubated at 37 °C. After 7 days incubation, 30 μL of alamar blue solution was added to each well, and the plate was re-incubated overnight. A change in color from blue (oxidized state) to pink (reduced) indicated the growth of mycobacteria because of the change of color due to respiration of active cells, and the MIC was defined as the lowest concentration of drug that prevented this change in color. Each reaction was carried out in triplicates. Pure chemical compounds used in this study can be found in different essential oils as major components including anise (*p*-anisaldehyde, *t-*anethole), bay (myrcene), blackcurrant (3-carene), camphor (camphor) caraway ((+)carvone), cinnamon (cinnamic acid, cinnamaldehyde), citronella (β-citronellol), clove (β-cariophylene, eugenol), cumin (*p-*cymene, cuminaldehyde), eucalyptus (eucalyptol), geranium (geraniol), holly oak (sabinene), lime ((+) limonene), mint (menthol), oregano (aarvacrol, thymol), tarragon (estragole), or they can be found in essential oils of plants from the *Pinaceae* family (β-pinene), of the *Lamiaceae* family (linalool) or present in several essential oils (α-terpinene, terpinolene). The evaluated compounds (terpenes and phenylpropanes) were acquired through the distributor Sigma-Aldrich (St. Louis, MI, USA) and their chemical structures are shown in Fig. 1.

### Cell lines, growth conditions and cytotoxic activity assays

The J774A mouse monocyte macrophage cell line was grown in RPMI medium (Sigma, R0883) supplemented with 10 % heat inactivated fetal bovine serum (FBS, Sigma-Aldrich), 1 % MEM-NEAA medium (Gibco, 11140–050) and a mixture of penicillin-streptomycin to 1 %. The cell culture was maintained at 37 °C with a partial atmosphere of 5 % CO_2_, the evaluations were carried out when 80–90 % confluence was reached.

In a 96 well plate 50,000 cells per well were deposited in a final volume of 100 μL, and incubated for 24 h at 37 °C in partial CO_2_ atmosphere to promote cell adhesion for an approximate 80 % confluence. Chemical compounds to be analyzed were diluted in DMSO; cytotoxic activity was evaluated by adding twofold serial dilutions of the compound to be analyzed, starting at a 1600 μg/mL concentration; the plates were further incubated (24 h). For cytotoxic evaluation, alamar blue (Sigma, R7017) solution was added (10 % *v/v*) and after 3 h, results were evaluated by determination of the change in color using a fluorimeter (590 nm). For negative control, cells were incubated with 500 μg/mL concentration of DMSO (Sigma, 472301), which was the highest concentration. Cytotoxic concentration (CC_50_) was determined using probit non-linear regression.

### Chemical descriptors characterization

Chemical structures of the compounds included in this work were analyzed with the Spartan 03 software [20] carrying out a conformational analysis of each molecule in gas phase using a mechanics force field SYBYL [21]. The minimum energy conformation was selected to obtain geometry optimization through a mechano-quantum calculation at level PM3 semi-empirical theory and numerical precision with minimal base [22]. Once the minimum energy geometries were obtained, the analytical frequency calculations were carried out for each stationary structure to verify if it was a minimum or a first order saddle point. Physicochemical, topological, constitutional, and charge descriptors were generated using the Dragon 5.0 program [23].

### Structure-antimycobacterial activity models

With all the biological activities (MIC for mycobacteria) and the calculated theoretical descriptors obtained, a QSAR study was carried out by generating genetic algorithms using the Mobydigs Software [24]. The quality of the model was considered statistically satisfactory based on the determination coefficient (R^2^) value, the leave-one-out cross-validated variance (Q^2^) and the F value of a given model. QSAR models were obtained using only structural descriptors in order to determine which structural arrangement and functional groups are important for the biological activity; in this case, for the antimycobacterial activity. QSAR models were also done based on all descriptors calculated from the molecular systems evaluated. Models were selected based on the four molecular properties that were identified as best related to the antimycobacterial activity.

### Molecular modeling and chemical reactivity analysis

Molecular systems were recalculated using the Gaussian 09 program [25] for the calculation of quantum mechanical descriptors related to reactivity. Geometry optimizations and frequency calculations were done by density functional theory (DFT) in aqueous phase, using the functional B3LYP which is a hybrid Hartree-Fock density functional theory (HF-DFT) functional that combines Becke’s three parameter exchange functional (B3) [26] and the correlation functional of Lee, Yang, and Parr (LYP) [27] in combination with the basis set 6-311G **.

Chemical reactivity descriptors: HOMO orbital and LUMO orbital energies; dipole moment, ionization potential, electron affinity, chemical potential, electronegativity, chemical hardness, chemical softness and aqueous solvation energy were obtained in aqueous phase from the energy calculations using Hartree-Fock (HF) with a 6-311G** basis set on optimized structures calculated with DFT:B3LYP/6-311**. The HOMO (highest occupied molecular orbital) and LUMO (lowest unoccupied molecular orbital) frontier orbitals, as well as the Koopmans theorem [28] were applied for the calculations of the chemical reactivity descriptors. All the properties obtained in aqueous phase were calculated considering a polarized Condutor-continuum model (CPCM) [29].

### Statistical analysis

Statistical analysis to reveal differences in antimicrobial activity of the chemical compounds analyzed was done by ANOVA test and differences within groups were done by Tukey test, with a 5 % significance level. Statistical analysis was done using the statistical software Minitab 17 [Computer software State College, PA].

## Results & Discussion

### *In vitro* potency of terpenes and phenylpropanes against *M. tuberculosis* and *M. bovis*

The major components of the essential oils were initially solubilized in pure ethanol, and the higher amount of the solvent was used as control to test for inhibitory effects, but no inhibitory effects were observed with the amount of solvent used. When considering MIC values of all compounds tested, a higher antimycobacterial activity was observed for *M. tuberculosis* strain as compared to *M. bovis* (average MIC for *M. tuberculosis* H37Rv was 22.78 μg/mL and average MIC for *M. bovis* AN5 was 32.07 μg/mL), and statistical analysis showed differences between the two strains. Thymol and carvacrol were the terpenes with higher antimycobacterial activity; thymol had MIC values of 0.78 and 2.02 μg/mL against strain of *M. tuberculosis* and *M. bovis* respectively. On the other hand, carvacrol presented MIC values of 2.02 and 5.20 μg/mL respectively for each mycobacteria tested. The antimycobacterial activity of thymol was grouped along with the activity of isoniazid and rifampicin by Tukey analysis (Table 1). Cinnamaldehyde and cinnamic acid were the most active phenylpropanes; cinnamic acid presented a MIC of 8.16 and 3.12 μg/mL for cinnamaldehyde; in both cases with the *M. tuberculosis* strain. In the case of *M. bovis,* cinnamic acid MIC was 7.29 μg/mL and MIC of cinnamaldehyde was 12.50 μg/mL. Caryophyllene and *p*-cymene were the terpenes with lower antimycobacterial activity.

**Table 1:** Antimycobacterial activity of terpenes and phenylpropanes against *M. tuberculosis* and *M. bovis*

Compounds		Antimycobacterial activity (μg/mL)	
		*M. tuberculosis*	*M. bovis*
		MIC	MIC
1	Cinnamic Acid	8.16 ± 3.32^FG^	7.29 ± 4.73^DE^
2	Camphor	41.66 ± 14.43^CD^	50.00 ± 0.00^B^
3	*p*-Anisaldehyde	4.16 ± 1.81^FG^	10.41 ± 3.61^DE^
4	*β*-pinene	10.41 ± 3.61^CDE^	41.66 ± 14.43^CDE^
5	3-Carene	16.66 ± 7.22^CDEF^	33.33 ± 14.43^CDE^
6	*p-*Cymene	91.66 ± 14.43 ^A^	91.66 ± 14.43 ^A^
7	*β*-Caryophyllene	100.00 ± 0.00 ^A^	100.00 ± 0.00 ^A^
8	Carvacrol	2.02 ± 0.88^FG^	5.20 ± 1.81^DE^
9	(+) Carvone	20.83 ± 7.22^CDE^	41.33 ± 15.01^BCDE^
10	Cinnamaldehyde	3.12 ± 0.00^EFG^	12.50 ± 0.00^DE^
11	*β*-Citronellol	6.25 ± 0.00^FG^	10.41 ± 3.61^DE^
12	Cuminaldehyde	10.41 ± 3.61^DEFG^	20.83 ± 7.22^CDE^
13	Estragole	20.83 ± 7.22^DEFG^	25.00 ± 0.00^BCDE^
14	Eucalyptol	41.66 ± 14.43^CDE^	41.66 ± 14.43^B^
15	Eugenol	25.00 ± 0.00^DEFG^	20.83 ± 7.22^BCD^
16	Geraniol	12.50 ± 0.00^EFG^	12.50 ± 0.00 ^CDE^
17	(+) Limonene	25.00 ± 0.00^CDE^	41.66 ± 14.43^BCD^
18	Linalool	33.33 ± 14.43^CDE^	41.66 ± 14.43^BC^
19	Menthol	41.66 ± 14.43 ^AB^	83.33 ± 14.43^B^
20	Myrcene	25.00 ± 0.00^CDEF^	33.33 ± 14.43^BCD^
21	Sabinene	33.33 ± 14.43^DEFG^	25.00 ± 0.00^BC^
22	*t-*Anethole	20.83 ± 7.22^BC^	58.33 ± 14.43^BCDE^
23	α-Terpinene	10.41 ± 3.61^DEFG^	20.83 ± 7.22^CDE^
24	Terpinolene	8.33 ± 3.61^CDEF^	33.33 ± 14.43^DE^
25	Thymol	0.78 ± 0.01^G^	2.02 ± 0.88^E^
TX	Isoniazid	0.26 ± 0.01^G^	0.16 ± 0.3^E^
TX	Rifampicin	0.60 ± 0.14^G^	0.50 ± 0.01^E^

Values are the average and standard deviation of triplicate assays. Superscripts correspond to clusters by similarity analysis done by Tukey mean analysis

Citronellol, geraniol and myrcene, are acyclic monoterpenes with structural similarities and MIC results were 6.25, 12.5 and 25 μg/mL for *M. tuberculosis* respectively. Geranylgeraniol and geranylgeranyl acetate have been identified as potent and selective inhibitors against *M. tuberculosis* with a MIC of 1.56 and 3.13 μg/mL respectively [30]. Linalool is an acyclic monoterpene with a tertiary hydroxyl group and has a lower antimycobacterial activity as compared to geraniol and citronellol. Results indicate that the primary hydroxyl group confers increased activity to the open chain terpenes. The importance of the hydroxyl group is also evident in the model structure-antimycobacterial activity against *M. bovis*.

Antimycobacterial activity assays using the Alamar Blue technique against *M. tuberculosis* and *M. bovis* showed that thymol and carvacrol (major constituents of the oregano essential oil) as well as cinnamic acid and cinnamaldehyde (constituent of the cinnamon essential oil) have antimycobacterial activities of therapeutic importance. The efficiency of the antimycobacterial activity of thymol and carvacrol has been reported previously [31]; also, the biological activity against mycobacteria has been reported for the complete essential oil [32, 33]. Cinnamic acid has been used as a synergist with drugs traditionally used in tuberculosis treatment [34].

The deleterious effect on structure and function of microbial membrane and cell wall, has been generally used to explain the antimicrobial action of the essential oils and their components especially monoterpenoids. It has been shown that monoterpenes are able to interact with phospholipid membranes, functioning as interstitial impurities in the ordered structure of the lipid bilayer [35] as a result of their lipophilic character; it can then be described the preference of terpenes to the microbial membrane structures [36]. The effects of specific components of some essential oil on the permeability of the outer membrane of Gram-negative bacteria have been demonstrated, showing that the absorption of the monoterpene is also determined by the permeability of the outer envelope of the microorganism. This hypothesis demonstrates a potential use of terpenes and phenylpropanes as antimycobacterial agents, since the mycobacteria cell wall is highly lipophilic due to the presence of mycolic acids. The lipophilicity of the major components of essential oils allows them to interact easily with the mycobacterial cell wall, with the consequent changes on cell permeability and microbial death.

### Cytotoxic activity of terpenes on macrophage J774A cell line

Carvacrol and thymol were analyzed to determine their cytotoxic activity, since both compounds demonstrated the higher antimycobacterial activity of all compunds tested. Due to its structural similarities, the cytotoxic activity of *p*-cymene and menthol were also evaluated, with the idea of being able to identify a structural relationship in the cytotoxic activity of these compounds. Results are shown in Table 2 and show a low cytotoxic activity of each compound as related to its antimicrobial activity. Thymol had a CC_50_ of 483.33 μg/mL while carvacrol eliminated 50 % of the macrophages at a concentration of 900 μg/mL. Menthol was the terpene with the lower cytotoxic activity. Although carvacrol and thymol presented cytotoxic activity, the CC50 concentration is much higher than the concentration needed for mycobacterial elimination (approximately 500 fold); the compounds can then, be considered as potential antimycobacterial agents.

**Table 2:** Cytotoxic concentrations of *p*-Cymene, carvacrol, menthol and thymol solutions on J774 macrophages

Compounds	Cytotoxic activity (μg/mL)	
	CC_50_	CC_90_
*p-*Cymene	1166.67 ± 44.34	2200.00 ± 86.60
Carvacrol	900.00 ± 86.60	1950.00 ± 107.44
Menthol	1450.00 ± 180.28	2600.00 ± 304.14
Thymol	483.33 ± 28.87	1133.33 ± 115.47

Values are the average and standard deviation of three assays

### QSAR models for antimycobacterial activity

The QSAR model for the H37Rv strain of *M. tuberculosis* that showed a statistical significance for structural properties included the following descriptors: number of total tertiary C(sp^3^) (nCt), number of terminal primary C (sp^2^) (nR = Cp), the number of phenolic groups (nArOH) and an inverse relationship with the number of ketone groups (nRCO). The best model is expressed in Eq. 1 and it demonstrates the importance of the phenolic groups and π bonds in the structures of major components of essential oils with antimycobacterial activity.

1 $$ \mathrm{LogMIC} = 0.1879(nCt) + 0.03630\left(nR = Cp\right) + 1.1729(nArOH)\ \hbox{--}\ 1.1159(nRCO)\ \hbox{--}\ 0.8750 $$

**Table:** 

*n* = 25	Q^2^ = 74.63	R^2^ = 80.23	F = 14.2	s = 0.245

Results of QSAR models of chemical constituents of essential oils for *M. bovis* AN5 showed that the number of conjugated carbons (nCconj), the number of phenolic groups (nArOH), the number of hydroxyl groups (OH) and the number of acceptor atoms of hydrogen bonds (nHAcc) were the most important structural descriptors in the activity. The best model is described in Eq. 2 and as with the H37Rv strain of *M. tuberculosis*, the model for *M. bovis* AN5 strain shows that the phenolic and hydroxyl groups are functional groups of biological importance.

2 $$ \mathrm{LogMIC} = 0.09324(nCconj) + 1.0900(nArOH) + 0.3395(nOH) + 0.2349(nHAcc)\ \hbox{--}\ 1.6318 $$

**Table:** 

*n* = 25	Q^2^ = 79.09	R^2^ = 82.11	F = 14.9	s = 0.186

Regarding molecular properties and antimycobacterial activity against H37Rv strain of *M. tuberculosis*, the best model included the following predictors: partition coefficient Octanol/Water (MLogP), molar volume (MV), absolute total charge (Qtot) and electron affinity (A). The model is expressed in the Eq. 3.

3 $$ \mathrm{LogMIC} = 0.4824(MLogP) + 0.0281(MV) - 0.01775(Qtot) - 0.1889(A) + 3.0240 $$

**Table:** 

*n* = 25	Q^2^ = 81.25	R^2^ = 91.69	F = 24.9	s = 0.106

**Fig. 2:**
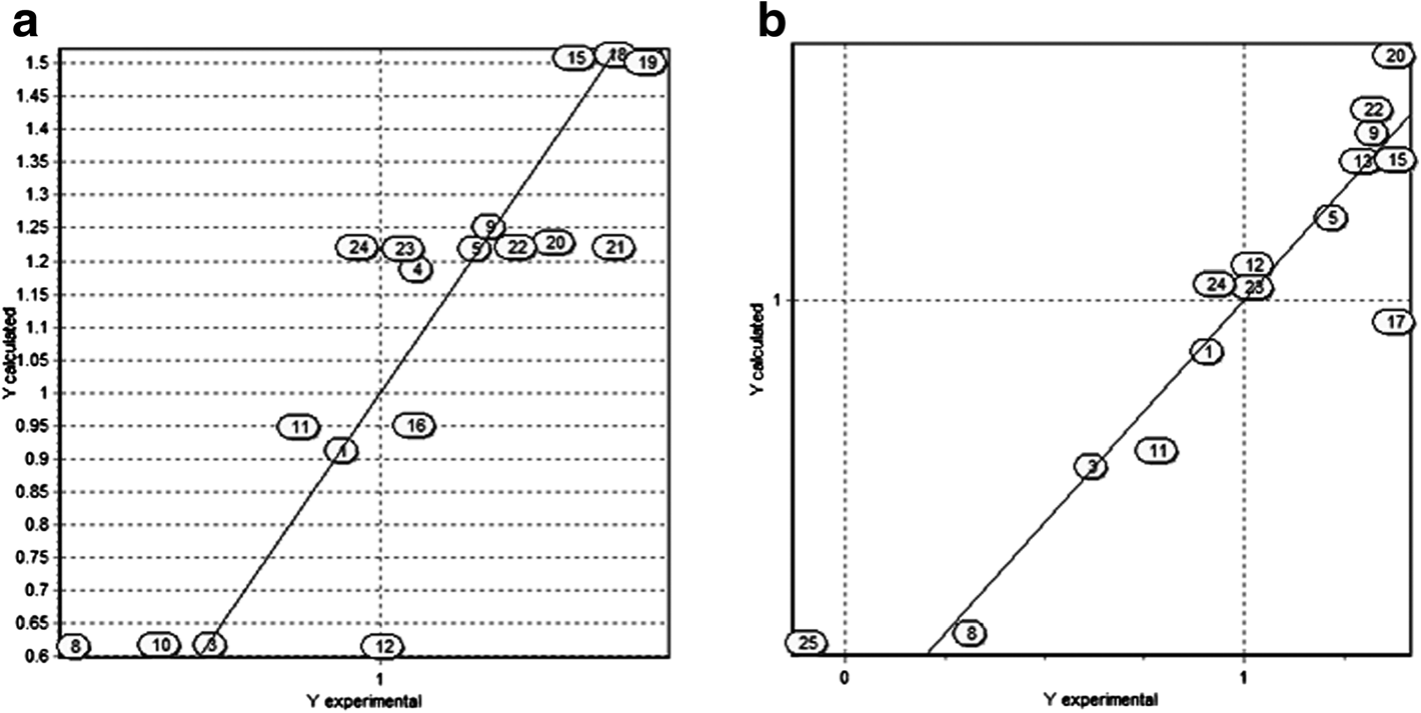
Predicted versus experimental antimycobacterial activity against *M. tuberculosis* for molecules in the training set used. **a** Structural descriptors model. **b** Molecular descriptors model

This model considers the Moriguchi octanol-water partition coefficient (MLogP) and molar volume (MV) descriptors as those with higher contribution to the antimycobacterial activity. A plot of the predicted activity versus experimental activity for molecules using a training set for models of *M. tuberculosis* is shown in Fig. 2. The statistics for other three QSAR models generated by analysis of genetic algorithms are included in Table 3.

**Table 3:** A summary of the statistics QSAR models for activity against *M. tuberculosis*

Parameter	Structure-activity relationship			Parameter	Property-Activity Relationship		
	Model 2	Model 3	Model 4		Model 2	Model 3	Model 4
n	25	25	25	n	25	25	25
Q^2^	70.50	69.25	67.25	Q^2^	81.25	79.13	78.16
R^2^	77.83	77.54	77.54	R^2^	87.69	87.08	87.08
F	12.3	12.1	12.1	F	24.9	23.6	23.6
s	0.260	0.261	0.261	s	0.160	0.164	0.164
Contributions (%)				Contributions (%)			
nCrt	0.1678	--	--	MlogP	0.4824	0.4559	0.4559
nCq	--	0.3001	--	MV	0.0281	0.0283	0.0283
nCrq	--	--	0.3001	E_LUMO_	0.1889	--	0.1877
nR = Cp	0.3635	0.3657	0.3657	Se	--	- 0.1144	- 0.1144
nArOH	1.0495	1.0570	1.0570	Qtot	- 0.0175	--	--
nRCO	– 1.1609	– 1.0026	– 1.0026	A	--	- 0.1877	--
Intercept	– 0.9395	– 0.9470	– 0.9470	Intercept	3.0240	2.2534	2.2534

*n* Number of systems evaluated, *Q* ^*2*^ The square of the coefficient of cross-validation, *R* ^*2*^ The square of the correlation coefficient, *s* standard deviation, *F* Fisher statistic

Physicochemical, structural, topological and constitutional descriptors: *nCrt* number of ring tertiary C (sp^3^), *nCq* number of total quaternary C (sp^3^), *nCrq* number of ring quaternary C (sp3), *nR = Cp* number of terminal primary C (sp^2^), *nArOH* number of phenolic groups, *nRCO* number of ketone groups, *E*_*LUMO*_ Energy LUMO orbital, *MlogP* Moriguchi octanol-water partition coeff., *MV* Molar Volume, *Se* sum of atomic Sanderson electronegativities (scaled on Carbon atom), *Qtot* total absolute charge), *A* Electron Affinity

Regarding molecular properties, the best QSAR model (Eq. 4) considers the full set of descriptors, being the four with higher biological significance the unipolarity (UNIP), the hydrophilicity (Hy), the molar volume (MV) and the dipole moment (m).

4 $$ \mathrm{LogMIC} = 0.07613\left(\mathrm{U} NIP\right)\ \hbox{--}\ 0.9780\ (Hy) + 0.2692\left(\mathrm{M}\mathrm{V}\right)\ \hbox{--}\ 0.2497\ (m) + 1.0095 $$

**Table:** 

*n* = 25	Q^2^ = 44.67	R^2^ = 78.49	F = 10.9	s = 0.201

**Fig. 3:**
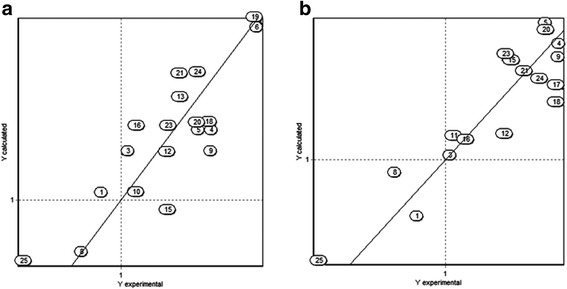
Predicted versus experimental antimycobacterial activity against *M. bovis* for molecules in the training set based. **a** Structural descriptors model. **b** Molecular descriptors model

A plot of the predicted activity versus experimental activity for molecules using a training set for *M. bovis* models is shown in Fig. 3. The statistics of other three QSAR models generated by analysis of genetic algorithms are shown in Table 4.

**Table 4:** A summary of the statistics of the QSAR models for activity against *M. bovis*

Parameter	Structure-activity relationship			Parameter	Property-Activity Relationship		
	Model 2	Model 3	Model 4		Model 2	Model 3	Model 4
n	25	25	25	n	25	25	25
Q^2^	76.94	75.12	72.31	Q^2^	70.25	70.64	69.51
R^2^	79.89	79.10	77.06	R^2^	76.89	74.43	72.54
F	12.7	12.3	10.9	F	10.0	8.7	7.9
s	0.198	0.201	0.210	s	0.209	0.219	0.227
Contributions (%)				Contributions (%)			
nCp	- 0.1381	--	- 0.0921	UNIP	0.1095	0.1052	0.0609
nCconj	0.0893	0.0557	0.0611	Hy	- 1.3558	- 1.3505	- 0.9442
nArOH	1.3088	1.0765	0.9820	AlogP	0.4974	--	--
nOH	0.6965	--	--	AlogP^2^	--	0.0831	--
nR = Ct	--	0.0968	--	E_HOMO_	--	--	- 0.2230
HAcc	--	0.3346	0.2500	m	- 0.2835	- 0.2653	- 0.1215
Intercept	- 1.2011	- 1.7189	- 1.5389	Intercept	0.0065	0.6590	2.3500

*n* Number of systems evaluated, *Q* ^*2*^ The square of the coefficient of cross-validation, *R* ^*2*^ The square of the correlation coefficient, *s* standard deviation, *F* Fisher statistic

Physicochemical, structural, topological and constitutional descriptors: *nCp* Number of terminal primary C (sp^3^), *nCconj* Number of non-aromatic conjugated C (sp^2^), *nArOH* Number of fenólico groups, *nOH* Number of hydroxyl groups, *nR = Ct* Number of aliphatic tertiary C(sp^2^), *HAcc* Number of acceptor atoms for H-bonds (N,O,F), *UNIP* Unipolarity, *AlogP* Ghose-Crippen octanol-water partition coeff., *AlogP* ^*2*^ Squared Ghose-Crippen octanol-water partition coeff., *E*_*HOMO*_ Energy of the HOMO orbital, *m* Dipole moment

**Table 5:** Theoretical descriptors of chemical reactivity for terpenes calculated by Hartree-Fock (HF) with a 6-311G**

Molecule	E_HOMO_	E_LUMO_	GapE	m	I	A	μ	χ	η	*S*	ΔG_solv_
	(eV)	(eV)	(eV)	(Debye)	(eV)	(eV)	(eV)	(eV)	(eV)	(eV)	(kcal/mol)
*p*-Cymene	−8.4628	3.8068	4.6560	0.0890	8.4628	−3.8068	2.3280	−2.3280	6.1348	0.1630	7.71
Carvacrol	−8.2859	3.9429	4.3431	1.9475	8.2859	−3.9429	2.1715	−2.1715	6.1144	0.1635	1.11
Menthol	−10.8438	5.7769	5.0669	2.4083	10.8438	−5.7769	2.5335	−2.5335	8.3104	0.1203	3.63
Thymol	−8.3104	3.8640	4.4465	1.8865	8.3104	−3.8640	2.2232	−2.2232	6.0872	0.1643	1.67

*E*_*HOMO*_ Energy of the HOMO orbital, *E*_*LUMO*_ Energy LUMO orbital, *m* Dipole moment, *GapE* E_LUMO_ - E_HOMO_ Gap energy, *I* Ionization potential, *A* Electron Affinity, *μ* Chemical potential *χ* Electronegativity, ƞ = Chemical hardness, *S* Chemical softness, *ω* Electrophilicity, *ΔG*_*solv*_ Aqueous solvation energy

Studies relating molecular properties of compounds and their antimycobacterial activity strengthen this hypothesis, since the descriptor octanol/water (MlogP) shows the higher contribution to the activity against *M. tuberculosis* in the QSAR model. For *M. bovis* QSAR model, the hydrophilicity (Hy) property exhibits the most important contribution, but the relationship is inverse with antimycobacterial activity.

The Molar Volume (MV) descriptor has been used to suggest a possible mechanism of action of the antimicrobial compounds in the solute-transfer process across biological membranes [37]. This descriptor is included in both models based on molecular properties, which suggest that the mechanism of action of terpenes and phenylpropanes might be related to an effect on the mycobacterial cell wall. QSAR studies of molecular structure proposes that the number of conjugated carbons (nCconj) as well as the number of terminal primary C(sp^2^) (nR = Cp) are important structural descriptors for the antimycobacterial activity, which can be related to the biological activity of aldehydes.

Regarding QSAR models for molecular properties and antimycobacterial activity, the models indicate that the partition coefficient octanol/water (MlogP) and molar volume (MV) descriptors are the major contributors for the biological activity. The chemical reactivity theoretical descriptors such as dipole moment (m) and electron affinity (A), were considered by the model as descriptors in inverse relation to the biological activity. The absolute total charge (Qtot) (a charge descriptor) was also considered in inverse relation to the biological activity.

Even though results of QSAR models suggests that the chemical compounds identified as major constituents of essential oils have as target structure of the antimicrobial activity the microbial cell wall, it is important to also consider other biological targets. It had been reported that thymol and carvacrol molecules have affinity for the chorismate mutase enzyme that is part of the shikimate pathway and is a key enzyme located at the branching point of the pathway [31]. Because of the importance of shikimic acid pathway for the synthesis of aromatic aminoacids, which is only found in Eubacteria and Protista organisms, it is important to be considered as a prime target for the development of new antimicrobial agents. Under this premise, it was decided to focus the study of chemical reactivity and cytotoxicity of thymol, carvacrol, *p*-cymene and menthol, compounds which have structural similarity and have different antimycobacterial activity.

### Chemical reactivity analysis of p-Cymene, menthol, carvacrol and thymol

**Fig. 4:**
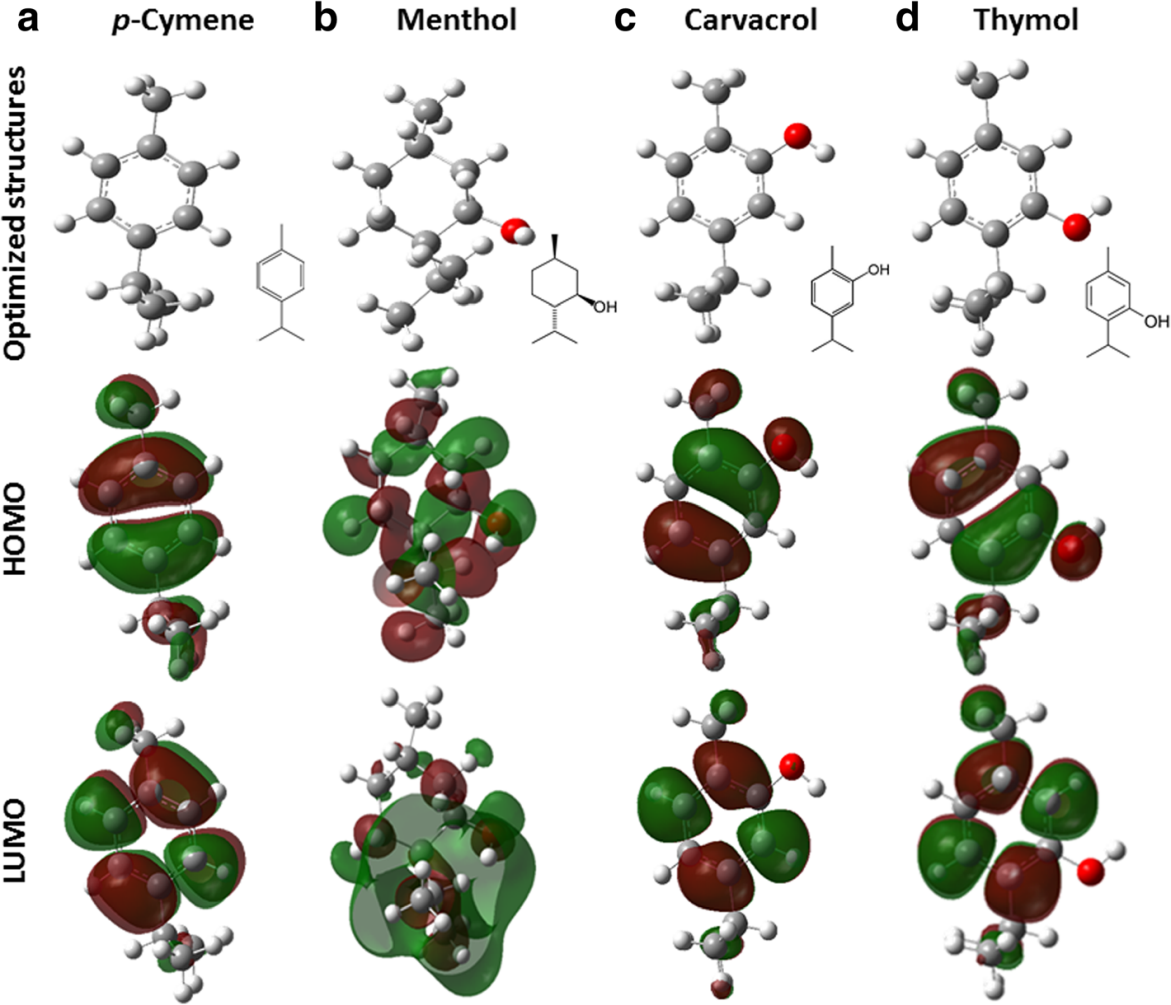
Molecular systems optimized calculated by DFT B3LYP/6-311G** level at theory and mapping of the frontier orbitals. **a** p-cymene **b** Menthol **c** Carvacrol **d** Thymol

A Chemical reactivity analysis was done for the compounds with MIC values of therapeutic importance (thymol and caravacrol), but *p*-cymene and menthol were included considering their structural similarity with the first two compounds. The Optimized structures and the contour plots for the HOMO and LUMO molecular orbitals are shown in Fig. 4.

The HOMO and LUMO molecular orbitals of *p-*cymene, carvacrol and thymol are located on the benzene rings, in the case of thymol and carvacrol molecules the HOMO orbital also includes oxygen atoms. The location of the HOMO and LUMO orbitals of menthol molecule have less symmetry, which could be associated with the absence of π bonds in the structure. Chemical reactivity analysis revealed that menthol shows the lowest energy calculated for the HOMO orbital of the four compounds analyzed, and presents the largest dipole moment and chemical hardness. On the other hand, *p-*cymene has the highest energy for the LUMO orbital and the lowest value for the dipole moment. Thymol and carvacrol showed intermediate values in most chemical reactivity descriptors (Table 5).

The most active compounds for both strains have phenolic groups in their chemical structure. There are several reports on the importance of the hydroxyl group present in thymol and carvacrol, and the authors describe this functional group as key for their antimicrobial activity [8]. This observation is supported by the QSAR models reported here, which consider the phenolic group (nArOH) as the structural descriptor of biological importance. The importance of the phenolic group is also observed by comparing the biological activities of thymol and menthol, since the difference is precisely the aromatic ring conformation. Menthol and thymol have the hydroxyl group in the same position but the menthol had lower antimicrobial activity, and also lower cytotoxic activity. Furthermore, the difference in activities between thymol and carvacrol resides in the hydroxyl group position relative to the larger aliphatic chain. Thymol has the hydroxyl group in meta position while carvacrol contains the hydroxyl group in the ortho position and the later has lower antimycobacterial and cytotoxic activity. On the contrary, Alokam et al. [26] reported an increased activity in carvacrol as compared to thymol. The elimination of the substituents of the aromatic ring in carvacrol has been shown to reduces the antimicrobial activity of the resulting compound against *S. aureus*; even when the hydroxyl group is replaced by an amino group, the activity is lost [38]. Since the elimination of the organic groups does not affect hydrophobicity, spatial structure, and solubility of the resulting compound as compared to carvacrol, it can be concluded that the hydroxyl group confers a special chemical features that add to the antimicrobial mode of action of carvacrol.

A large number of useful molecular descriptors, related to the physical properties and chemical reactivity of molecules, can be derived from the information available from molecular orbitals. The energies of the highest occupied molecular orbital and the lowest unoccupied molecular orbital, E_HOMO_ and E_LUMO_ respectively belong to the most popular quantum mechanical descriptors used. The HOMO orbital is used as an indicator of the highest electron density area, so that these zones exhibit a favorable region to be attacked by electrophiles, while a reactive or nucleophilic compound will be attracted to areas with lower electron density indicated for LUMO orbital.

The HOMO orbital is mainly located on the benzene groups (Fig. 4) but in thymol and carvacrol, this orbital is positioned above the phenolic group. The Menthol molecule presents a less symmetrical distribution of HOMO orbital and shows the lowest energy of this orbital (E_HOMO_); of the four compounds analyzed, menthol was the system with highest chemical hardness (η). Furthermore, menthol has a higher dipole moment (m) and ionization potential (I). Thymol and carvacrol were the compounds with lower free energy of solvation. Cymene has the smaller dipole moment (m) and the higher solvation free energy (ΔG_solv_), and is therefore, the less water-soluble compound. These results demonstrate that the lipophilicity alone is not the responsible for the antimycobacterial activity, but this activity is also linked to the electronic characteristics of the phenolic group.

## Conclusion

The antimycobacterial activity of terpenes and phenylpropanes that are present in diverse essential oils was analyzed by MIC for two mycobacterial strains. Molecules with higher antimycobacterial activity showed low cytotoxicity for macrophages and are candidates for further testing in *in vivo* models. The description of the molecular properties and the structural characteristics responsible for antimycobacterial activity of the tested compounds, were used for the development of mathematical models of quantitative structure-activity relationship (QSAR). The identification of molecular and structural descriptors provide insight into the mechanisms of action of the active molecules; the information provided can be used for the design of new chemical structures, that could be synthetized as potential new antimycobacterial agents.
